# EZH2 as a major histone methyltransferase in PDGF-BB-activated orbital fibroblast in the pathogenesis of Graves’ ophthalmopathy

**DOI:** 10.1038/s41598-024-57926-x

**Published:** 2024-04-04

**Authors:** Sopita Visamol, Tanapat Palaga, Preamjit Saonanon, Vannakorn Pruksakorn, Nattiya Hirankarn, P. Martin van Hagen, Willem A. Dik, Sita Virakul

**Affiliations:** 1 Medical Microbiology, Interdisciplinary Program, Graduate School, Chulalongkorn University, Bangkok, Thailand; 2 Department of Microbiology, Faculty of Science, Chulalongkorn University, Bangkok, Thailand; 3 Department of Ophthalmology, Faculty of Medicine, Chulalongkorn University, Bangkok, Thailand; 4 Department of Microbiology, Faculty of Medicine, Center of Excellence in Immunology and Immune Mediated Disease, Chulalongkorn University, Bangkok, Thailand; 5 Laboratory Medical Immunology, Department of Immunology, Erasmus MC, University Medical Center, Rotterdam, The Netherlands; 6 Division of Clinical Immunology, and Immunology, Department of Internal Medicine, Erasmus MC, University Medical Center, Rotterdam, The Netherlands

**Keywords:** Histone lysine methyltransferases, EZH2, H3K27me3, Graves’ ophthalmopathy, Orbital fibroblast, Mechanisms of disease, Inflammation, Target identification, Autoimmune diseases, Epigenetics, Preclinical research, Autoimmunity

## Abstract

Graves’ ophthalmopathy (GO) is an extra-thyroidal complication of Graves’ disease which can lead to vision loss in severe cases. Currently, treatments of GO are not sufficiently effective, so novel therapeutic strategies are needed. As platelet-derived growth factor (PDGF)-BB induces several effector mechanisms in GO orbital fibroblasts including cytokine production and myofibroblast activation, this study aims to investigate the roles of histone lysine methyltransferases (HKMTs) in PDGF-BB-activated GO orbital fibroblasts by screening with HKMTs inhibitors library. From the total of twelve selective HKMT inhibitors in the library, EZH2, G9a and DOT1L inhibitors, DZNeP, BIX01294 and Pinometostat, respectively, prevented PDGF-BB-induced proliferation and hyaluronan production by GO orbital fibroblasts. However, only EZH2 inhibitor, DZNeP, significantly blocked pro-inflammatory cytokine production. For the HKMTs expression in GO orbital fibroblasts, PDGF-BB significantly and time-dependently induced *EZH2*, *G9a* and *DOT1L* mRNA expression*.* To confirm the role of EZH2 in PDGF-BB-induced orbital fibroblast activation, *EZH2* silencing experiments revealed suppression of PDGF-BB-induced collagen type I and α-SMA expression along with decreasing histone H3 lysine 27 trimethylation (H3K27me3) level. In a more clinically relevant model than orbital fibroblast culture experiments, DZNeP treated GO orbital tissues significantly reduced pro-inflammatory cytokine production while slightly reduced *ACTA2* mRNA expression. Our data is the first to demonstrate that among all HKMTs EZH2 dominantly involved in the expression of myofibroblast markers in PDGF-BB-activated orbital fibroblast from GO presumably via H3K27me3. Thus, EZH2 may represent a novel therapeutics target for GO.

## Introduction

Graves’ ophthalmopathy (GO) is an extra-thyroidal complication found in 25–50% of Graves’ disease patients^27^. Upper eyelid retraction, edema, erythema of periorbital tissue, conjunctivitis, limitation of extraocular movement, proptosis and fibrosis are common symptoms of GO^27^. Severe symptoms such as corneal ulcers and compressive optic neuropathy leading to loss of vision were also found in some GO patients^27^.

Orbital fibroblasts are central cellular components in the pathogenesis of GO where they are the main contributors to orbital inflammation and tissue expansion^1,2,27^. Several factors, including thyroid stimulating hormone receptor (TSHR) stimulatory autoantibodies (thyroid stimulating antibodies (TSAb)), have been identified as activators of orbital fibroblasts in GO pathogenesis^2^. Platelet-derived growth factor (PDGF)-BB is also proposed to represent an important activator of orbital fibroblast activity in GO since it enhances orbital fibroblast proliferation, production of pro-inflammatory cytokines and hyaluronan, adipogenesis and TSHR expression that makes these orbital fibroblasts more susceptible to activation with TSAb^1,3^.

Current treatment regime for GO is not optimally effective and can even cause potentially negative effects on the patients^27^. Recently, teprotumumab, an anti-IGF-1 receptor monoclonal antibody, was approved as novel treatment for GO patients; however, around 30% of GO patients do not respond adequately to teprotumumab^28^. Therefore, new therapeutic strategies are needed to effectively treat GO.

Epigenetic mechanisms including histone modifications, non-coding RNAs, and DNA methylation play important roles in controlling gene expression^29^. So far data on epigenetic mechanisms in GO orbital fibroblasts are limited^4,5^. Recently, we and other groups demonstrated that DNA methylation and histone deacetylation involved in regulating orbital fibroblast activities in GO patients^6–8^. In addition, histone methylation, a process that involves the transfer of methyl groups to lysine or arginine residues on the histone tails, represents an important histone modification that is catalyzed by the enzymes histone lysine methyltransferases (HKMTs) and protein arginine methyltransferases (PRMTs)^9,29^. As studies showed crosstalk of methylation processes on both DNA and histone and different pre-clinical models showed the efficacy of inhibitors targeting these enzymes in several fibrotic diseases^9–15,29^. Thus, the discovery of HKMTs functions that control fibroblast activities in patient-derived primary fibroblasts may reveal potential novel targets for therapy. Therefore, this study explores whether aberrant regulation of HKMT activity contributes to PDGF-BB-induced orbital fibroblast activation in GO.

## Results

### EZH2, G9a and DOT1L inhibition reduced PDGF-BB-induced GO orbital fibroblast proliferation and hyaluronan production

**Figure 1:**
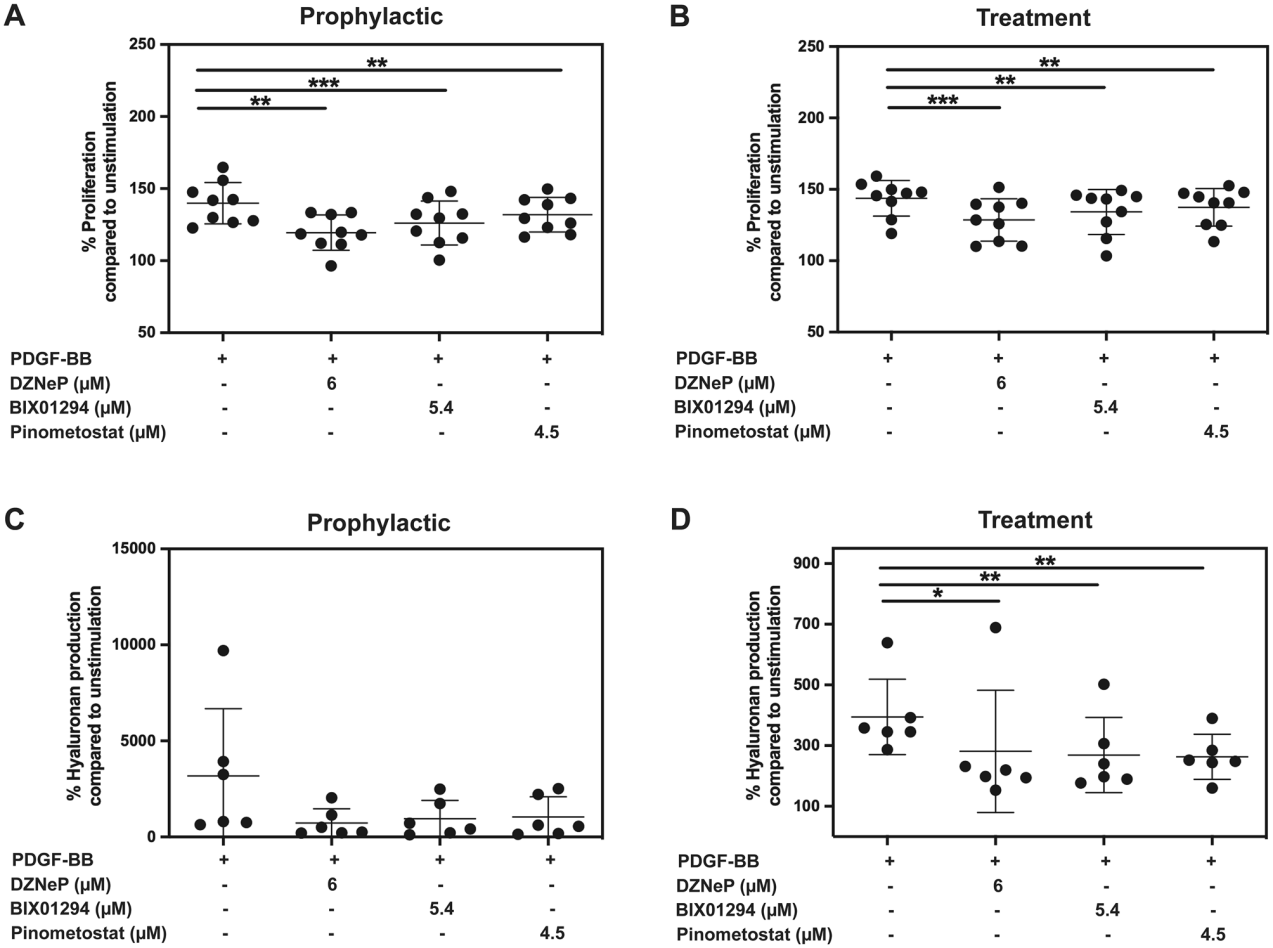
EZH2, G9a and DOT1L inhibition reduced PDGF-BB-induced orbital fibroblasts proliferation and hyaluronan production. (**A**) GO orbital fibroblasts (n = 9) were pre-incubated with selective HKMT inhibitors for 24 h and then stimulated with PDGF-BB (50 ng/ml) in the presence or absence of selective HKMT inhibitors. (**B**) GO orbital fibroblasts (n = 9) were stimulated with PDGF-BB (50 ng/ml) and simultaneously treated with selective HKMT inhibitors. After 24 h, proliferation was determined by methylene blue staining. (**C**) GO orbital fibroblasts (n = 6) were pre-incubated with selective HKMT inhibitors for 24 h and then stimulated with PDGF-BB (50 ng/ml) in the presence or absence selective HKMT inhibitors. (**D**) For treatment setting, the orbital fibroblasts (n = 6) were stimulated with PDGF-BB (50 ng/ml) and selective HKMT inhibitors for 24 h. Afterwards, supernatant was collected, and hyaluronan levels were measured by ELISA. Each dot represents the orbital fibroblast strain from one individual and horizontal bars represent the mean values ± standard deviation (SD). Data were analyzed using the paired Student’s *t*-test. ***, ** and * represent a *p*-value of < 0.001, < 0.01 and < 0.05, respectively, compared to PDGF-BB stimulation.

To investigate the role of HKMTs in PDGF-BB-induced orbital fibroblast activation, selective HKMTs inhibitors from Epigenetics Compound Library, at different concentrations, were screened. None of the tested HKMTs inhibitors were toxic to orbital fibroblasts (Supplementary Fig. S1), nor inhibited basal proliferation of the orbital fibroblasts (Supplementary Fig. S2A). To obtain the maximum pharmacological effect of the inhibitors, orbital fibroblasts (n = 9) were pre-treated (prophylactic setting) with the different HKMTs inhibitors. Inhibition of EZH2, G9a and DOTL1 with the highest concentrations of their inhibitors DZNeP (6 µM), BIX01294 (5.4 µM) and Pinometostat (4.5 µM), respectively, all significantly inhibited PDGF-BB-induced orbital fibroblast proliferation (Fig. 1A). The effect of DZNeP, BIX01294 and Pinometostat on PDGF-BB-induced orbital fibroblast proliferation was also explored in an experimental set-up considered more representative of a treatment condition. This revealed that when these HKMTs inhibitors were co-added with PDGF-BB to the orbital fibroblasts they did significantly abrogate PDGF-BB-induced proliferation (Fig. 1B). Therefore, only EZH2, G9a and DOT1L inhibitors were subsequently evaluated for their roles during orbital fibroblast activation as other HKMTs including EZH1, SETD7, SETD8, G9a/GLP, COMT and MLL were not interfered with their inhibitors, including CPI-360, EI1, CPI-169, PFI-2 HCI, UNC0379, A-366, Entacapone, MI-2 (for MLL with IC50 of 446 nM) and MM-102 (for MLL with IC50 of 0.4 µM), respectively (data not shown).

Next, we explored the effect of DZNeP, BIX01294 and Pinometostat on PDGF-BB-induced hyaluronan production by orbital fibroblasts. At the concentrations used for proliferation assay, none of the tested HKMTs inhibitors affected basal hyaluronan production by the orbital fibroblasts (Supplementary Fig. S2B). Pre-incubation with DZNeP, BIX01294 or Pinometostat did not significantly reduce PDGF-BB-induced % hyaluronan production by orbital fibroblasts (Fig. 1C) but did significantly reduce hyaluronan production from an average of 7048 ng/ml from PDGF-BB stimulated condition to 2582, 3045 and 3222 ng/ml, respectively (data not shown). However, when DZNeP, BIX01294 or Pinometostat were simultaneously added with PDGF-BB to the orbital fibroblasts these HKMT inhibitors significantly reduced PDGF-BB-induced hyaluronan production (Fig. 1D).

### EZH2 inhibition blocked PDGF-BB-induced pro-inflammatory cytokine production by orbital fibroblasts in both prophylactic and treatment settings

**Figure 2:**
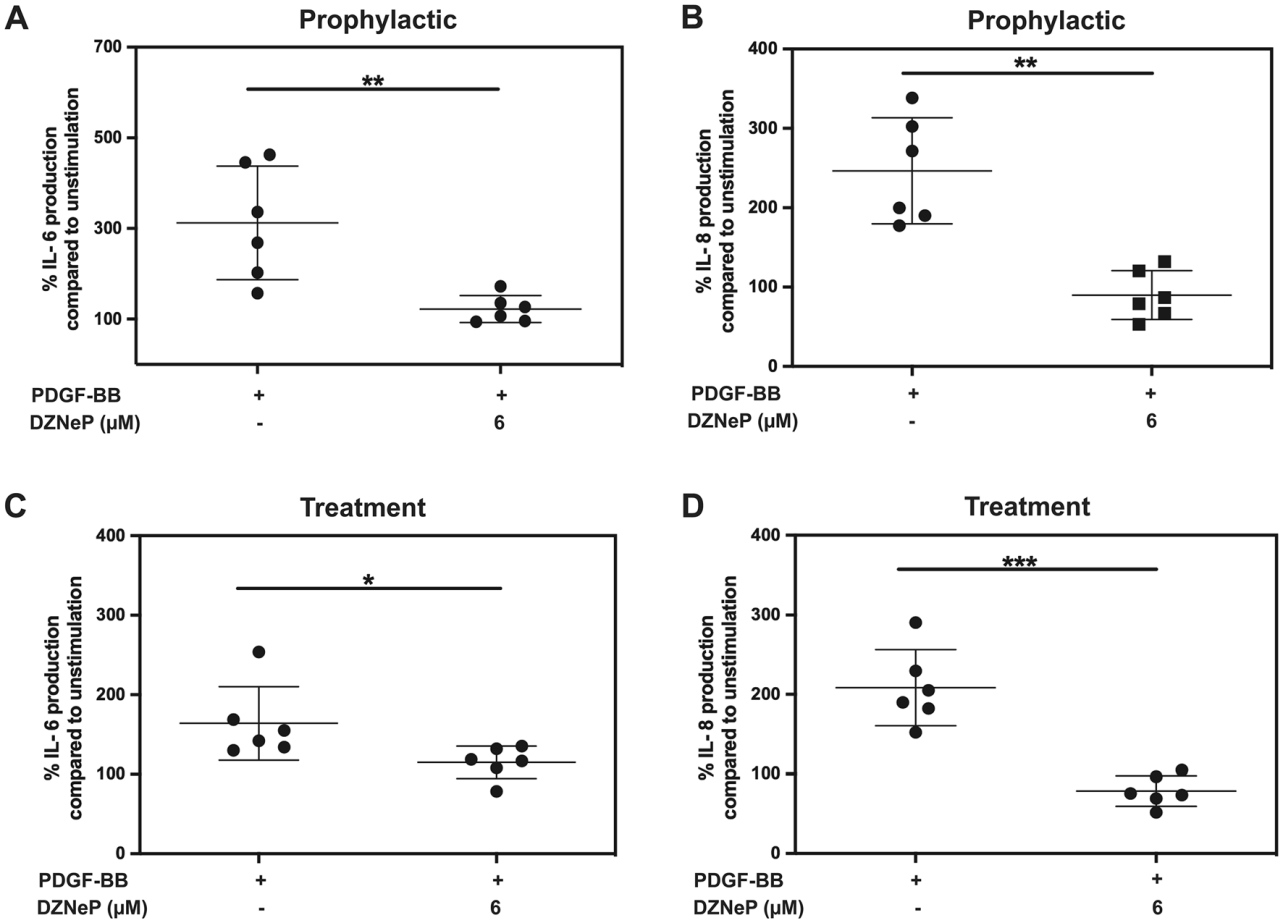
EZH2 inhibition blocked PDGF-BB-induced pro-inflammatory cytokine production by orbital fibroblasts in both prophylactic and treatment settings. (**A**, **B**) GO orbital fibroblasts (n = 6) were pre-incubated with DZNeP for 24 h and then stimulated with PDGF-BB (50 ng/ml) in the presence or absence of DZNeP. (**C**, **D**) The orbital fibroblasts (n = 6) were stimulated with PDGF-BB (50 ng/ml) and treated with DZNeP at the same time without pre-incubation step. After 24 h, supernatant was collected, and IL-6 (**A**, **C**) and IL-8 (**B**, **D**) levels were measured by ELISA. Each dot represents the orbital fibroblast strain from one individual and horizontal bars represent the mean values ± standard deviation (SD). Data were analyzed using the paired Student’s *t*-test. ***, ** and * represent a *p*-value of < 0.001, < 0.01 and < 0.05, respectively, compared to PDGF-BB stimulation.

To investigate the role of HKMTs in PDGF-BB-induced pro-inflammatory cytokine production by orbital fibroblasts, the effect of DZNeP, BIX01294 and Pinometostat on PDGF-BB-induced IL-6 and IL-8 production were determined. None of the tested HKMT inhibitors reduced basal IL-6 and IL-8 production by the orbital fibroblasts (Supplementary Fig. S2C,D). Pinometostat and BIX01294 did not inhibit PDGF-BB-induced IL-6 nor IL-8 production, both in the experimental prophylactic and treatment setting (Supplementary Figs. S3 and 4). Pre-treatment of orbital fibroblasts with DZNeP significantly reduced PDGF-BB-induced IL-6 and IL-8 production (Fig. 2A,B). Moreover, DZNeP also significantly suppressed PDGF-BB-induced IL-6 and IL-8 production when co-added with PDGF-BB, with an inhibitory effect of ~ 30% and 60%, respectively (Fig. 2C,D).

### PDGF-BB induced HKMTs expression in orbital fibroblasts

**Figure 3:**
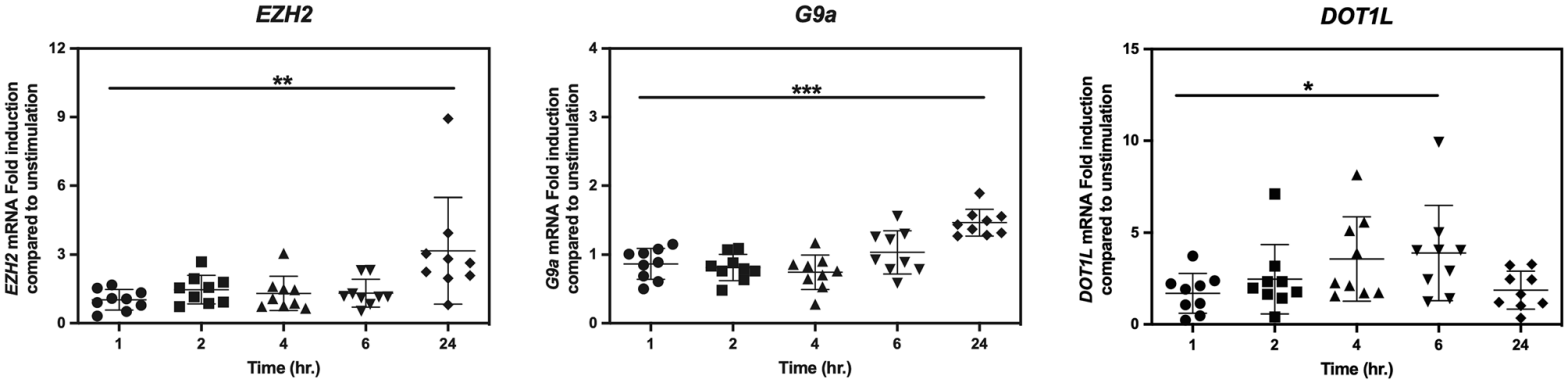
PDGF-BB induced HKMTs expression in orbital fibroblasts. GO orbital fibroblasts (n = 9) were stimulated with PDGF-BB (50 ng/ml) for 1, 2, 4, 6 or 24 h. Transcription levels were determined by real-time PCR and normalized to the *ABL*. Fold induction at all time points were further compared to unstimulated condition. Each dot represents the orbital fibroblast strain from one individual and horizontal bars represent the mean values ± standard deviation (SD). Data were analyzed using the paired Student’s *t*-test. ***, ** and * represent a *p*-value of < 0.001, < 0.01 and < 0.05, respectively, compared to PDGF-BB stimulation at one hour.

*EZH2, G9a* and *DOT1L* mRNA expression were investigated, and the data showed that there was no difference in basal mRNA expression levels of *EZH2, G9a* and *DOT1L* between orbital fibroblasts obtained from GO patients and controls (Supplementary Fig. S5). On the other hand, PDGF-BB significantly and time-dependently enhanced mRNA expression of the HKMTs in GO orbital fibroblasts. *EZH2* and *G9a* mRNA expression were significantly elevated after 24 h of PDGF-BB exposure with *EZH2* expressed at the highest level, while *DOT1L* mRNA level was significantly elevated after 6 h of PDGF-BB stimulation after which it declined again at 24 h (Fig. 3).

### Silencing EZH2 reduced PDGF-BB-induced ECM production by orbital fibroblasts

**Figure 4:**
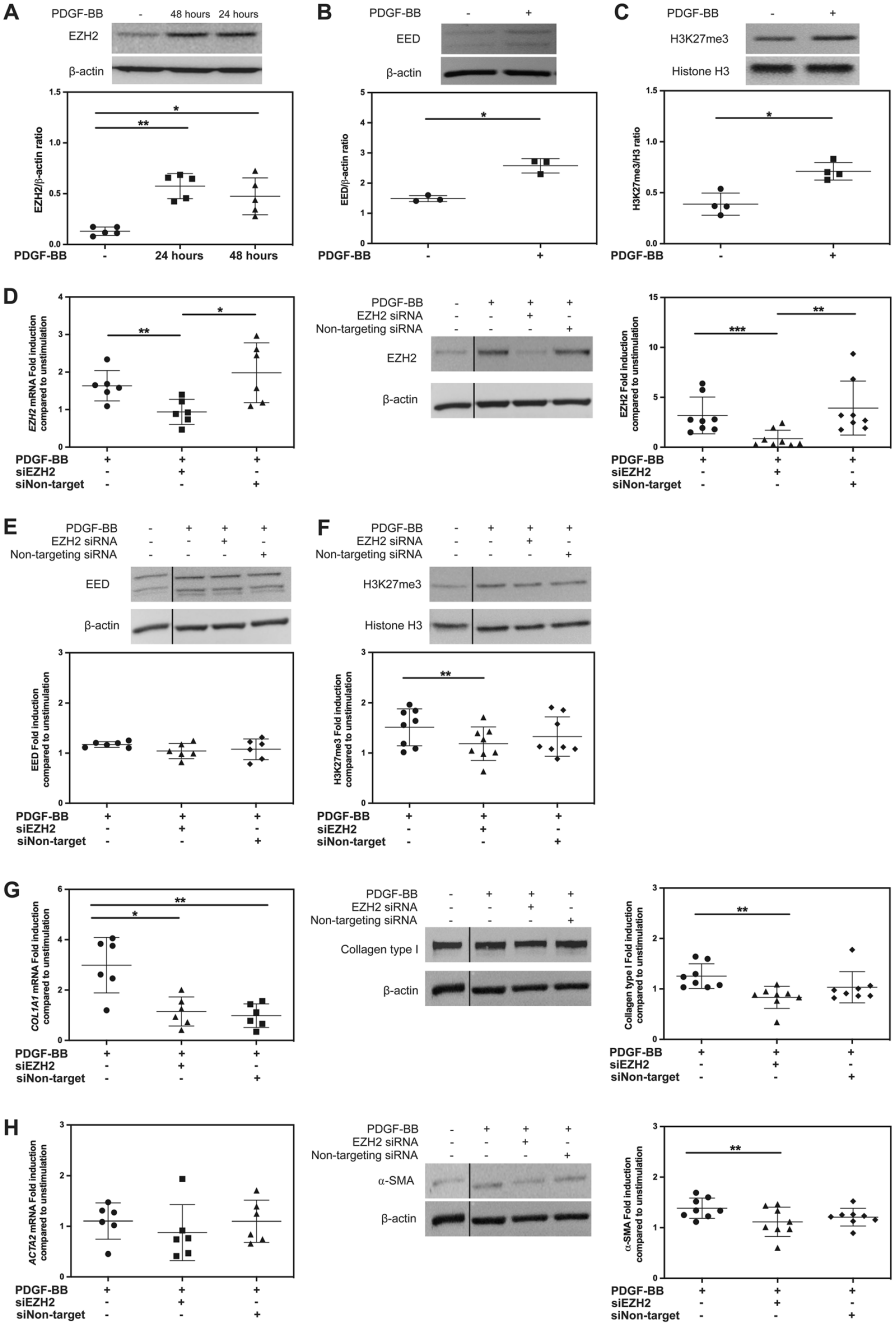
Silencing *EZH2* reduced PDGF-BB-induced ECM production by orbital fibroblasts. The protein level of EZH2 (**A**), EED (**B**) and H3K27me3 (**C**) were determined by western blot analysis in orbital fibroblasts (n = 3–5) from GO patients stimulated with PDGF-BB (50 ng/ml) for 24 and/or 48 h. Data were analyzed using the paired Student’s *t*-test. ** and * represent a *p*-value of < 0.01 and < 0.05, respectively, compared to the unstimulated condition. GO orbital fibroblasts (n = 6–8) were transfected with *EZH2* siRNA or non-targeting siRNA for 48 h and then stimulated with PDGF-BB (50 ng/ml). *EZH2* (**D**, left panel) mRNA expression was determined by real-time PCR and normalized to *ABL* after 24 h of PDGF-BB stimulation. The level of EZH2 (**D**, right panel) protein expression were determined by western blot analysis and normalized to β-actin expression after 48 h of PDGF-BB stimulation. After 48 h of PDGF-BB stimulation, the level of EED (**E**) protein expression were determined by western blot analysis and normalized to β-actin expression. The level of H3K27me3 protein expression (**F**) was determined by western blot analysis and normalized to histone H3 expression. *COL1A1* (**G**, left panel) and *ACTA2* (**H**, left panel) mRNA expression were determined by real-time PCR and normalized to *ABL* after 24 h of PDGF-BB stimulation from six different orbital fibroblast strains. The level of collagen type I (**G**, right panel) and $\alpha$-SMA (**H**, right panel) protein expression were determined by western blot analysis and normalized to β-actin expression after 48 h of PDGF-BB stimulation. The Western blot data (middle panel) is from a representative sample of one orbital fibroblast, while the data on the right panel is from eight different orbital fibroblast strains. Cropped blots are displayed with dividing lines. The uncropped blots are displayed in Supplementary Fig. S9. Fold induction at all time points were further calculated compared to unstimulated condition. Each dot represents the orbital fibroblast strain from one individual and horizontal bars represent the mean values ± standard deviation (SD). Data were analyzed by using paired Student’s *t*-test. ***, ** and * represent a *p*-value of < 0.001, < 0.01 and < 0.05, respectively, compared to unstimulated condition, PDGF-BB stimulation alone or with non-targeting siRNA.

Since the data strongly supports a dominant role for EZH2 in PDGF-BB-activated orbital fibroblast activation, we further explored the effect of PDGF-BB on EZH2 protein expression in orbital fibroblasts. PDGF-BB significantly enhanced orbital fibroblast EZH2 protein expression after 24 and 48 h of stimulation (Fig. 4A). EZH2 works in the polycomb repressive complex 2 (PRC2) which also consists of another core subunit required for PRC2 catalytic activity to stimulate EZH2-induced H3K27me3 histone lysine methyltransferase activity called embryonic ectoderm development (EED)^16^. Therefore, EED protein level was also determined upon PDGF-BB stimulation. EED protein expression significantly increased after 48 h of PDGF-BB exposure (Fig. 4B), which was also accompanied by increased methylation of H3K27me3 (Fig. 4C).

Subsequently, *EZH2* expression was silenced by specific *EZH2* siRNA. *EZH2* siRNA significantly (*P* < 0.05), yet not totally, reduced basal *EZH2* mRNA expression (~ 50% reduction compared to non-targeting siRNA; Supplementary Fig. S6A). *EZH2* siRNA did not affect *EZH1* mRNA expression (Supplementary Fig. S6B). *EZH2* siRNA significantly decreased basal EZH2 protein level compared to non-targeting siRNA, while *EZH2* siRNA did not reduce basal EED protein expression nor basal H3K27me3 level in the orbital fibroblasts (Supplementary Fig. S6C–E). On the other hand, PDGF-BB stimulation alone significantly induced EZH2 at both mRNA and protein expression (1.7- and 3.1-fold, respectively) in *EZH2* siRNA transfection experiments, while *EZH2* siRNA transfection significantly reduced PDGF-BB-induced EZH2 at both mRNA and protein expression in all samples (~ 55% and ~ 81% reduction compared to non-targeting siRNA condition, respectively) (Fig. 4D). In contrast, *EZH2* siRNA transfection did not reduce EED protein expression in all conditions (Fig. 4E), while PDGF-BB-induced H3K27me3 was significantly reduced in the presence of *EZH2* siRNA compared to PDGF-BB stimulation alone (Fig. 4F).

For the effect of *EZH2* silencing on orbital fibroblast activation markers, *EZH2* siRNA transfection significantly reduced PDGF-BB-induced-*COL1A1* but not *ACTA2* mRNA expression compared to PDGF-BB stimulation alone (Fig. 4G,H). However, *EZH2* siRNA transfection significantly reduced PDGF-BB-induced collagen type I and $\alpha$-SMA protein expression compared to PDGF-BB stimulation alone (Fig. 4G,H). On the other hand, neither *Ki67*, *IL6*, *HAS2* mRNA nor IL-6 and hyaluronan production level was affected by *EZH2* siRNA (Supplementary Fig. S7A–C).

### Effect of EZH2 inhibition in whole orbital tissue from inactive GO patients

**Figure 5:**
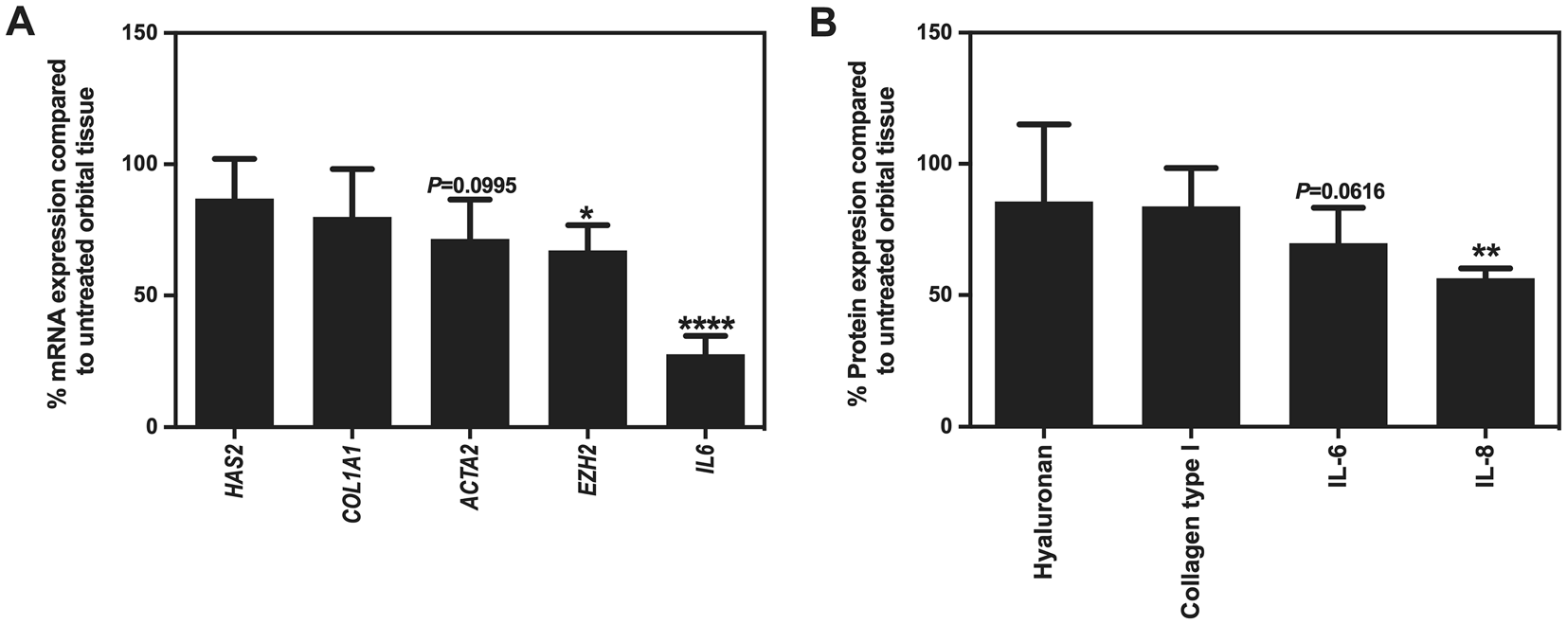
Effect of EZH2 inhibition in whole orbital tissue from inactive GO patients. GO orbital tissues (n = 3–10) were cultured with DZNeP (EZH2 inhibitor) for 24 h. *HAS2*, *COL1A1*, *ACTA2, EZH2* and *IL6* mRNA expression level from the orbital tissues were determined by real-time PCR, normalized to the *ABL,* and calculated % expression relative to the untreated tissues (**A**). Hyaluronan, collagen type I, IL-6 and IL-8 levels were measured by ELISA and calculated % expression relative to the untreated tissues (**B**). Horizontal bars represent the mean values, error bars indicate the standard error of the mean. Data were analyzed using the paired *t*-test. ****, ** and * represent a *p*-value of < 0.0001, < 0.01 and < 0.05, respectively, compared to untreated tissue. *P* represents a *p*-value compared to untreated tissue.

We further explored the role of EZH2 in cultured orbital tissue explants from GO patients which is more clinically relevance than orbital fibroblast culture experiments. DZNeP was used at 6 µM, a non-cytotoxic concentration comparable to the in vitro experiments (Supplementary Fig. S8). DZNeP significantly reduced *EZH2* and *IL6* mRNA expression by approximately 30% and 70%, respectively (Fig. 5A). On the other hand, DZNeP did not affect *COL1A1*, *ACTA2* and *HAS2* mRNA expression while *Ki67* mRNA expression was undetectable in orbital tissue. In line with the mRNA observations, DZNeP did not affect collagen type I and hyaluronan levels in treated orbital tissues while IL-6 and IL-8 levels did decline (*P* = 0.0616 and *P* < 0.05, respectively, Fig. 5A,B).

## Discussion

PDGF-BB stimulates several orbital fibroblast effector functions in the pathogenesis of GO^1^. As orbital fibroblasts are well recognized to be different in their embryonic origin from fibroblasts of other anatomical regions, epigenetically-regulated phenotype responses could be distinct from fibroblasts from other anatomical locations or diseases and thus warrants investigation^17^. Therefore, we aimed to reveal for the first time that HKMT activity is involved in orbital fibroblast effector functions induced by PDGF-BB.

The functional roles of each HKMTs were validated with their selective inhibitors and EZH2 activities showed the predominant role in controlling PDGF-BB-induced orbital fibroblasts activation which could be diminished by its inhibitor, DZNeP (Figs. 1 and 2). From these observations, we speculated that G9a and DOTL1 might play important role during proliferation and hyaluronan production rather than cytokine production. Moreover, our data is in the same line with other fibrotic models that EZH2 inhibition suppressed fibrogenesis^10,18–23^ and pro-inflammatory cytokine levels^20,23^ and that DOT1L and G9a inhibition attenuated inflammation^11,13^. However, from all the data on HKMTs inhibitors in this study, blocking certain HKMTs did reduce PDGF-BB-induced orbital fibroblast activation although the inhibition was never complete. Consequently, we speculate that cooperation between the different HKMTs as well as other histone modifications like HDAC4 as previously reported may very well be involved in establishing the pro-mitogenic, pro-inflammatory and pro-fibrotic effect of PDGF-BB in orbital fibroblasts^6^. Therefore, future studies into these complex regulations are certainly warranted.

*EZH2* silencing experiments showed significant reduction of PDGF-BB-induced methylation level on H3K27me3 in orbital fibroblasts (Fig. 4F), confirming that siRNA transfection affected both a reduction in EZH2 expression level per se and also the histone methyltransferase activity which is more specific than the inhibitory effect from the inhibitor. Although we could not exclude the fact that EZH1 was involved in the histone methylation, still our current data support a role of PDGF-BB in inducing de novo EZH2 expression which results in increased methylation level of H3K27me3. Another alternative approach to confirm EZH2 mechanistic activities in GO pathogenesis could be directed at other PRC2 subunits such as EZH1, EED and SUZ12^24^ which requires further studies. Next, *EZH2* silencing significantly decreased PDGF-BB-induced *COL1A1* mRNA and collagen type I and $\alpha$-SMA protein expression (Fig. 4G,H). Consistent with our findings, Wasson CW, *et al*. reported that EZH2 inhibition reduced collagen and $\alpha$-SMA expression in scleroderma dermal fibroblasts^21^. On the contrary, knockdown of EZH2 did not reduce PDGF-BB-induced *Ki67*, *HAS2* and *IL6* mRNA expression nor hyaluronan and IL-6 production (Supplementary Fig. S7). With the limitation in primary orbital fibroblast cell number, we were unable to perform ChIP-seq analysis to extensively identify target genes regulated by EZH2/H3K27me3. Therefore, further studies are needed to clarify the mechanism of EZH2 as increased understanding into these mechanisms may provide rationales to target EZH2 in the treatment for GO patients.

The discrepancies between results obtained from orbital fibroblast cultures experiments and ex vivo orbital tissue experiments on hyaluronan inhibition might be because insufficiently high concentrations of DZNeP were reached in the orbital tissue samples with our approach. Moreover, DZNeP could inhibit EZH2 in other cell populations in orbital tissues environment and change their phenotypes such as endothelial cells, macrophages and T cells which result in the interference in the total response of certain gene and protein expression level^10,20,25^. In addition, other histone modifications could play roles in this complex microenvironment which limit the efficiency of EZH2 blockage. Nevertheless, this finding is a promising approach to treat GO by inhibiting EZH2 activities.

Considering our previous observation that the DNA methylation pattern of orbital fibroblasts from inactive GO are more similar to that of control orbital fibroblasts than that of orbital fibroblasts from active GO^7^, our current data on the mRNA expression levels of *EZH2, G9a* and *DOT1L* were also comparable between orbital fibroblasts from inactive GO patients and controls (Supplementary Fig. S5). As we did not measure EZH2 expression and H3K27me3 level in orbital tissue and did not include active GO to compare with inactive GO and controls in our current study, we cannot draw a conclusion on roles and dynamics of these HKMTs during GO progression. This is a limitation of our current study and warrants further studies.

In summary, our data is the first to report that PDGF-BB induces the expression of the HKMTs *G9a*, *DOT1L* and *EZH2* in orbital fibroblasts from GO and EZH2 is important in PDGF-BB-induced proliferation, cytokine and hyaluronan production in GO orbital fibroblasts. Therefore, treatment targeting EZH2 may have a potential therapeutic benefit in treating GO patients.

## Materials and methods

### Isolation of orbital fibroblasts from orbital tissues

Orbital fibroblasts were isolated from orbital tissue of GO patients (n = 25) at an inactive stage of disease who underwent orbital decompression surgery at King Chulalongkorn Memorial Hospital (Bangkok, Thailand). GO patient characteristics are shown in Supplementary Table S1. Control orbital tissues (n = 6) were obtained from individuals without thyroid or inflammatory disease that underwent cosmetic surgery. Informed consent was obtained from all the patients. This study was in accordance with Declaration of Helsinki. Approval for the study was given by the Institutional Review Board of the Faculty of Medicine (Protocol number 401/ 61), Chulalongkorn University (Bangkok, Thailand). Primary orbital fibroblasts were used for experiments between the 2nd and 6th passages as described previously^6^. With the variation on the total number of cells isolated from each orbital tissues, orbital fibroblast from different individuals were used from n = 3–16 per experiment.

### Effect of HKMT inhibition on orbital fibroblast proliferation

GO orbital fibroblasts were plated at 5 × 10^3^ cells/well onto 96-well plates with DMEM containing 1%FBS and antibiotics overnight. For the screening experiments, orbital fibroblasts were pre-incubated with selective HKMT inhibitors from Epigenetics Compound Library (Catalog No. L1900, Selleck Chemicals, Inc., TX) for 24 h and stimulated with 50 ng/ml of PDGF-BB (Biolegend Inc., USA), either with or without the HKMTs inhibitors for another 24 h. For further experiments on orbital fibroblast activation, only EZH2, G9a and DOT1L inhibitors were used as shown in Supplementary Table S2. In addition, orbital fibroblasts were exposed to PDGF-BB and HKMTs inhibitors simultaneously for 24 h in an experimental set-up more representative for disease treatment as previously reported^6^. Then, orbital fibroblast proliferation was determined based on uptake and subsequent release of methylene blue dye, as described previously^26^. Percentages of proliferation were calculated by the following formula: (test sample/negative control) *100.

### Effect of HKMT inhibitions on ECM production and pro-inflammatory cytokine by orbital fibroblasts

GO orbital fibroblasts were plated at 1 × 10^5^ cells/well in 12-well plates in DMEM containing 1% FBS and antibiotics overnight. The effect of HKMTs inhibitors for both prophylactic and treatment settings were investigated as described above. Supernatant was collected and hyaluronan (Cat# DY3614, R&D system Inc., USA), IL-6 (Cat# 430504, RRID:AB_2935703) and IL-8 (Cat# 431504, RRID:AB_2935704) (Biolegend Inc., USA) levels were measured by ELISA according to the manufacturer’s protocol. Percentages of ECM production and pro-inflammatory cytokine by orbital fibroblasts were calculated by the following formula: (test sample/unstimulated condition) *100.

### HKMTs mRNA expression

Orbital fibroblasts from GO (n = 9–16) and control (n = 6) were plated at 4 × 10^5^ cells/well onto 6-well plates with DMEM containing 1%FBS and antibiotics overnight. Orbital fibroblasts were stimulated with PDGF-BB (50 ng/ml) for 1, 2, 4, 6 and 24 h. Total RNA was isolated using GenElute™ Total RNA Purification Kit (Sigma-Aldrich, USA) and was converted into cDNA using iScript™ cDNA Synthesis Kit according to the manufacturer’s protocol (Bio-Rad Inc., USA). Messenger RNA expression levels of the HKMTs were determined with SsoAdvanced™ Universal Probes Supermix (Bio-Rad Inc., USA) by real-time PCR (CFX96 Touch™ Real-Time PCR Detection System). Gene expression level were normalized to the *ABL* as previously reported^6^. Primer and probe combinations are shown in Supplementary Table S3.

### EZH2, EED, H3K27me3, collagen type I and $\boldsymbol{\alpha }$-SMA expression in orbital fibroblasts

GO orbital fibroblasts were seeded at 3 × 10^5^ cells/well into 6-well plates in DMEM containing 1% FBS and antibiotics overnight. Orbital fibroblasts were stimulated with PDGF-BB (50 ng/ml) for 48 h as previously reported^6^. Then, protein expression was analyzed by Western blot with Amersham ECL Western Blotting Kit (GE Healthcare Life Sciences Inc., USA) as described previously^6^. All the antibodies are shown in Supplementary Table S4 (Ezh2 Cat#5246, RRID:AB_10694683, EED Cat#85322, RRID:AB_2923355, COL1A1 Cat#72026, RRID:AB_2904565, α-Smooth Muscle Actin Cat#19245, RRID:AB_2734735, GAPDH Cat#5174, RRID:AB_10622025, Histone H3 Cat#4499, RRID:AB_10544537, Tri-Methyl-Histone H3 (Lys27) Cat#9733, RRID:AB_2616029, β-Actin Cat#4970, RRID:AB_2223172 and mouse anti-rabbit IgG (HRP-conjugate) secondary antibody Cat#5127 RRID:AB_10892 860) (Cell Signaling Technology, USA). Then, protein expression levels were determined by Alliance Q9 Chemiluminescence Imaging System (Uvitec. Inc., UK). The intensity of each band was quantified by using ImageJ software. Protein expression levels were normalized to the β-actin or GAPDH. Each conditions were then calculated as fold induction compared to unstimulated condition as previously reported^6^.

### *EZH2* silencing in orbital fibroblasts

GO orbital fibroblasts were seeded at 3 × 10^5^ cells/well into 6-well plates in antibiotic-free medium overnight. Cells were transfected with 25 nM ON-TARGETplus Human *EZH2* siRNA or ON-TARGETplus Non-targeting Control siRNAs with DharmaFECT 1 Transfection Reagents (Dharmacon Inc., USA) according to the manufacturer’s protocol. After 48 h of silencing, the orbital fibroblasts were stimulated with PDGF-BB. Gene expression levels were determined by real-time PCR after 24 h for *EZH2, EZH1, COL1A1, ACTA2, Ki67, IL6, HAS2 and ABL* gene. Primer and probe combinations are shown in Supplementary Table S3. Moreover, protein expression levels were measured after 48 h of PDGF-BB stimulation, as described above.

### EZH2 inhibition in whole orbital tissue from GO patients

GO orbital tissues (n = 3–10) which represent an ex vivo experiment was performed in 24-well plates as previously reported^6^. Briefly, the tissues were incubated overnight in DMEM/1% FBS in presence or absence of DZNeP (EZH2 inhibitor; 6 µM) as previously reported^6^. Total RNA was isolated with RNeasy Mini Kit (QIAGEN, Germany) and converted into cDNA using iScript™ Reverse Transcription Supermix for RT-qPCR according to the manufacturer’s protocol (Bio-Rad Laboratories, US). Gene expression levels were determined by real-time PCR as described above. Supernatant was collected for LDH cytotoxicity, hyaluronan, collagen type I, IL-6 and IL-8 measurement by ELISA. Percentages of mRNA and protein expression were calculated by the following formula: (treated orbital tissues/untreated orbital tissues) *100.

### Statistical analyses

Data are presented as the mean ± standard deviation (SD)and analyzed by 1-way ANOVA and paired Student’s *t*-test for all GO orbital fibroblast experiment or unpaired *t*-test in the experiment that compare GO with control orbital fibroblasts by GraphPad Prism 9.

### Supplementary Information

Supplementary Information.

## Data Availability

All data generated or analysed during this study are included in this published article and its supplementary information files.
