# Supplementary material for: EZH2 as a major histone methyltransferase in PDGF-BB-activated orbital fibroblast in the pathogenesis of Graves’ ophthalmopathy
Source: Sci Rep. 2024 Apr 4;14:7947. doi: 10.1038/s41598-024-57926-x (PMC10994939; doi:10.1038/s41598-024-57926-x)
Supplement: Supplementary file 1 — Supplementary Information. [file 41598_2024_57926_MOESM1_ESM.pdf]

Supplementary Materials for

# EZH2 as a Major Histone Methyltransferase in PDGF-BB-activated Orbital Fibroblast in the pathogenesis of Graves' Ophthalmopathy

Sopita Visamol[1,i], Tanapat Palaga[2], Preamjit Saonanon[3], Vannakorn Pruksakorn[3], Nattiya Hirankarn[4], P. Martin van Hagen[4,5,6], Willem A. Dik[5] and Sita Virakul[2,ii,*]

*Corresponding Author: Sita Virakul, PhD

E-mail: sita.v@chula.ac.th

**The PDF file includes:**

Supplementary figures S1-S9

Supplementary Tables S1-S4

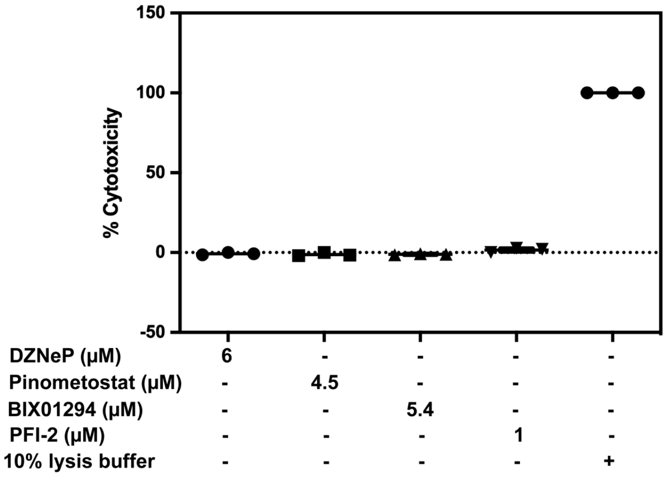


## Supplementary figure S1.

**%Cytotoxicity of HKMT inhibitors on orbital fibroblasts.** GO orbital fibroblasts (n=3) were incubated with histone methyltransferase inhibitors at their maximal concentration and supernatants were collected for LDH cytotoxicity assay.

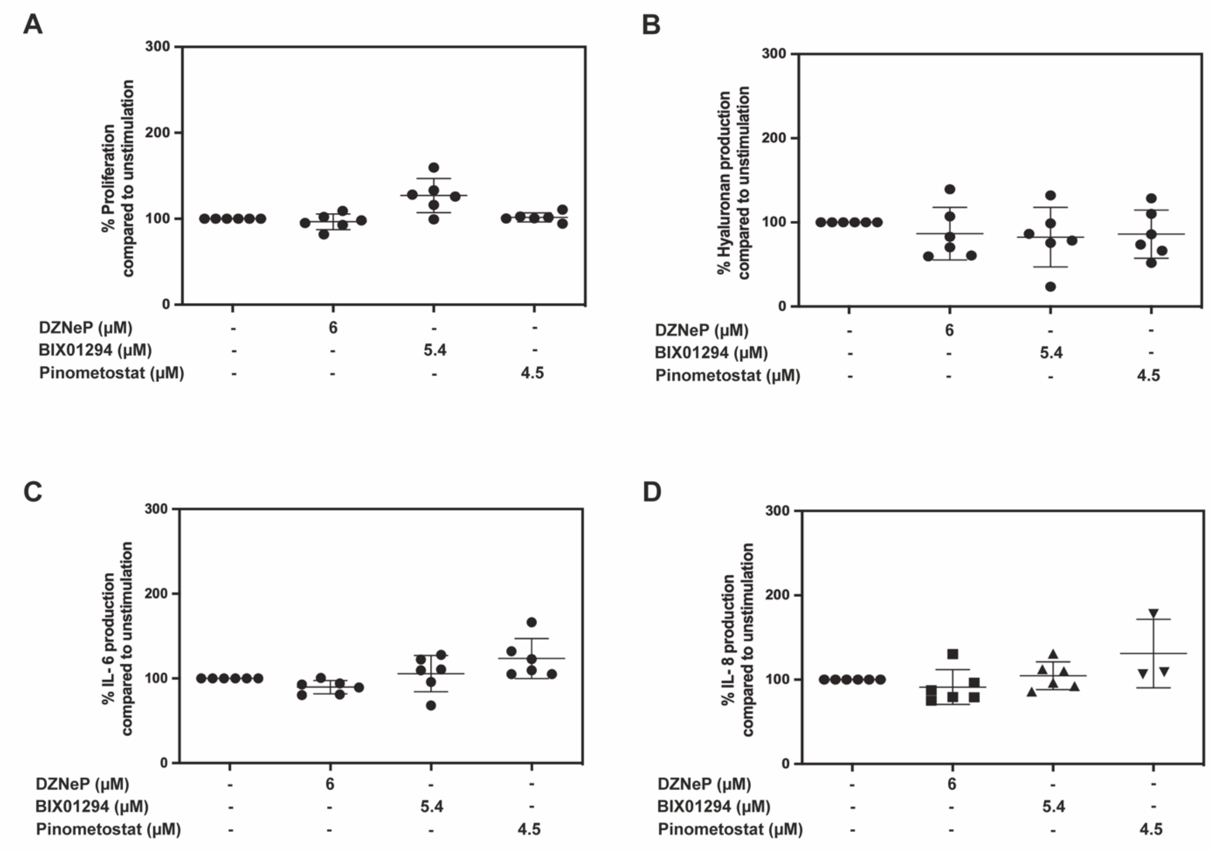


**Supplementary figure S2**.

**The effect of HKMT inhibitors on basal orbital fibroblast proliferation, hyaluronan and cytokine production. (A)** GO orbital fibroblasts (n=3-6) were pre-incubated with a histone methyltransferase inhibitor for 24 hours. After 24 hours, proliferation was determined by methylene blue staining. GO orbital fibroblasts were pre-incubated with a histone methyltransferase inhibitor for 24 hours and then stimulated with PDGF-BB (50 ng/ml) in the presence or absence specific HKMT inhibitors. After 24 hours, supernatant was collected, hyaluronan **(B)** and IL-6 **(C)** and IL-8 **(D)** levels were measured by ELISA. Each dot represents the orbital fibroblast strain from one individual and horizontal bars represent the mean values ± standard deviation (SD).

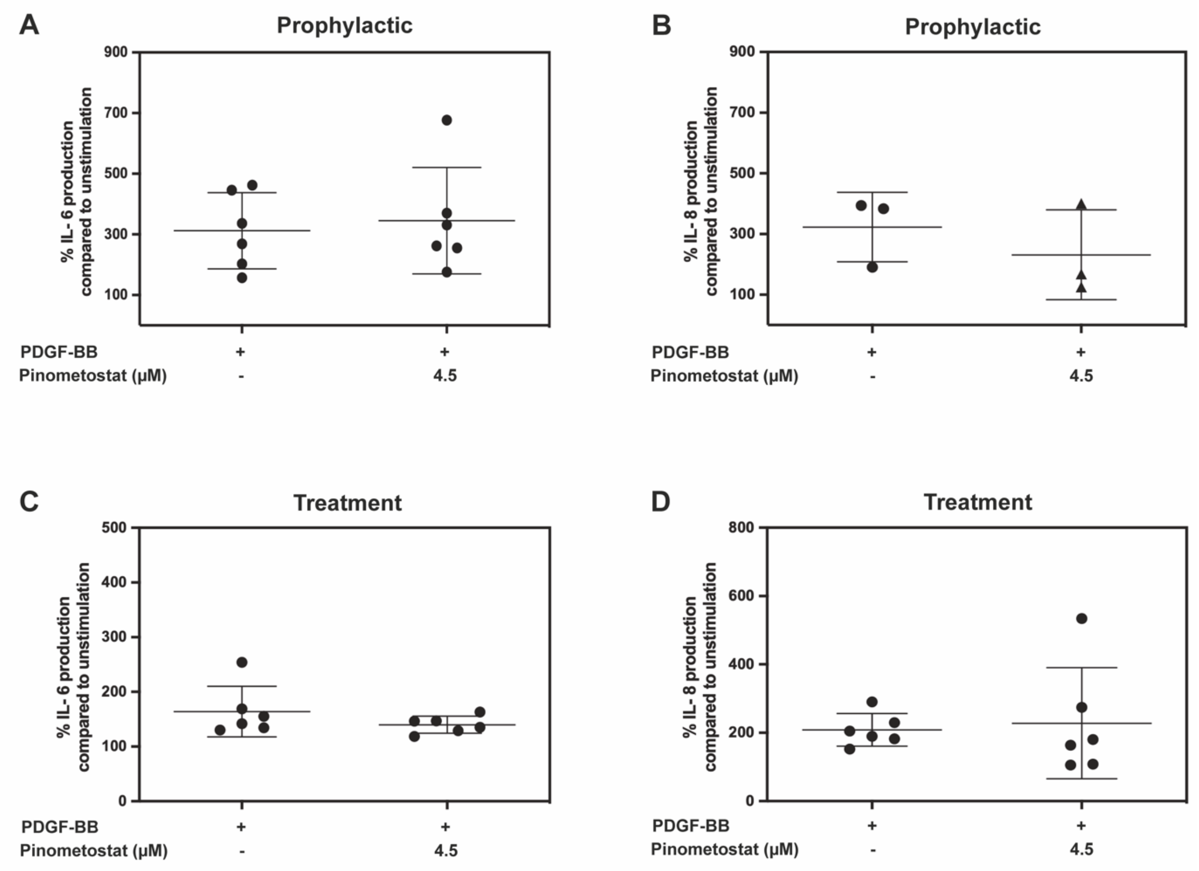


**Supplementary figure S3.**

**DOT1L inhibition with Pinometostat in PDGF-BB-induced pro-inflammatory cytokine production. (A, B)** GO orbital fibroblasts (n=3-6) were pre-incubated with Pinometostat for 24 hours and then stimulated with PDGF-BB (50 ng/ml) in the presence or absence of Pinometostat. **(C, D)** The orbital fibroblasts (n=6) were stimulated with PDGF-BB (50 ng/ml) and treated with Pinometostat at the same time without pre-incubation step. After PDGF-BB stimulation following 24 hours, supernatant was collected, and IL-6 **(A, C)** and IL-8 **(B, D)** levels were measured by ELISA. Each dot represents the orbital fibroblast strain from one individual and horizontal bars represent the mean values ± standard deviation (SD).

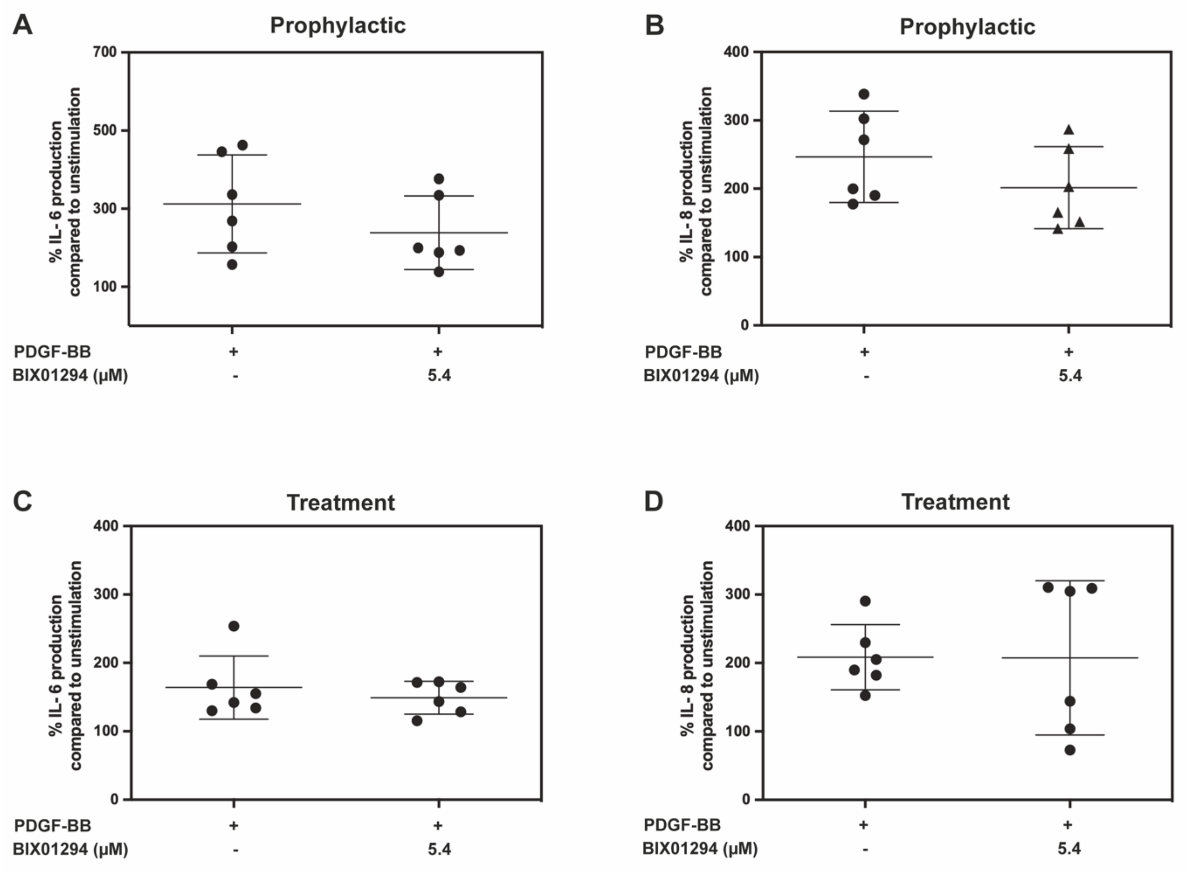


**Supplementary figure S4.**

**G9a inhibition with BIX01294 in PDGF-BB-induced pro-inflammatory cytokine production. (A, B)** GO orbital fibroblasts (n=6) were pre-incubated with BIX01294 for 24 hours and then stimulated with PDGF-BB (50 ng/ml) in the presence or absence of BIX01294. **(C, D)** The orbital fibroblasts (n=6) were stimulated with PDGF-BB (50 ng/ml) and treated with BIX01294 at the same time without pre-incubation step. After PDGF-BB stimulation following 24 hours, supernatant was collected, and IL-6 **(A, C)** and IL-8 **(B, D)** levels were measured by ELISA. Each dot represents the orbital fibroblast strain from one individual and horizontal bars represent the mean values ± standard deviation (SD).

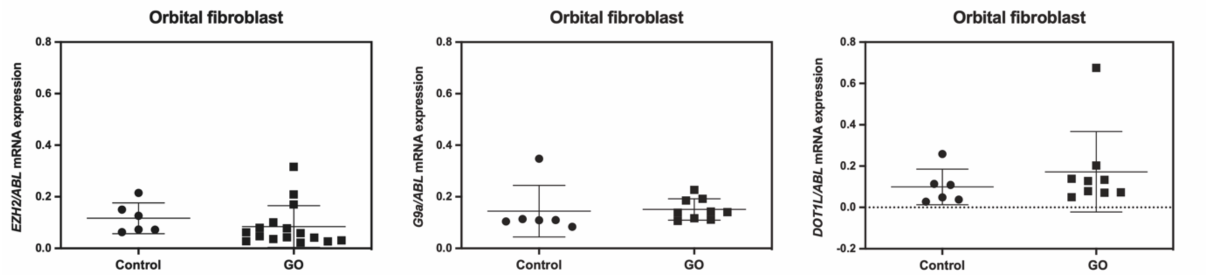


**Supplementary figure S5.**

***EZH2*, *G9a* and *DOT1L* mRNA expression in orbital fibroblasts isolated from healthy control and GO.** Total RNA from healthy control (n=6) and inactive GO (n=9-16) orbital fibroblasts were extracted, converted to cDNA and measured *EZH2, G9a* and *DOT1L* expression level by real-time PCR. Each dot represents the orbital fibroblast strain from one individual and horizontal bars represent the mean values ± standard deviation (SD).

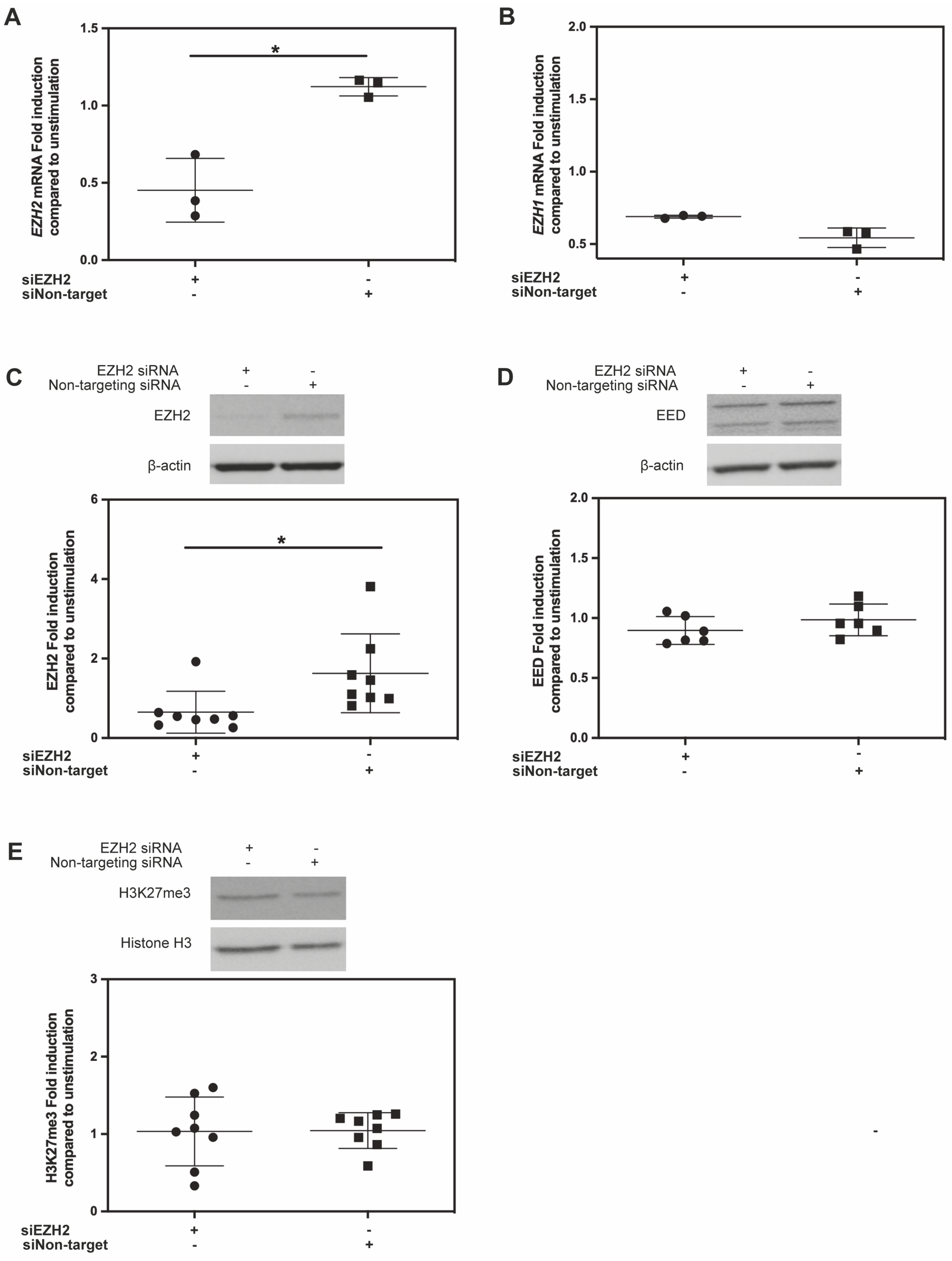
A
B
C
D
E
EZH2 mRNA Fold induction compared to unstimulation
EZH1 mRNA Fold induction compared to unstimulation
EZH2 Fold induction compared to unstimulation
EED Fold induction compared to unstimulation
H3K27me3 Fold induction compared to unstimulation
siEZH2
siNon-target
EZH2 siRNA
Non-targeting siRNA
EZH2
β-actin
EED
H3K27me3
Histone H3
*
0.0
0.5
1.0
1.5
2.0
0
1
2
3
4
6
+
-

**Supplementary figure S6**.

**The effect of *EZH2* siRNA on basal *EZH1* and *EZH2* mRNA, EZH2, EED and H3K27me3 protein expression in orbital fibroblasts.** GO orbital fibroblasts (n=3) were transfected with *EZH2* siRNA or non-targeting siRNA for 48 hours. The level of *EZH2* **(A)** and *EZH1* **(B)** mRNA expression was determined by real-time PCR and normalized to the *ABL*. * represent a p-value of <0.05, compared to non-targeting siRNA condition. GO orbital fibroblasts (n=6-8) were transfected with *EZH2* siRNA or non-targeting siRNA for 48 hours and then the level of EZH2 **(C)** and EED **(D)** protein expression was determined by western blot analysis and normalized to β-actin expression. The level of H3K27me3 protein expression **(E)** was determined by western blot analysis and normalized to histone H3 expression. The original blot images are shown in Supplementary fig. S9. Fold induction at all time points were further compared to unstimulated condition. Each dot represents the orbital fibroblast strain from one individual and horizontal bars represent the mean values ± standard deviation (SD). Data were analyzed using the paired Student's *t*-test. * represent a p-value of <0.05 compared to non-targeting siRNA**.**

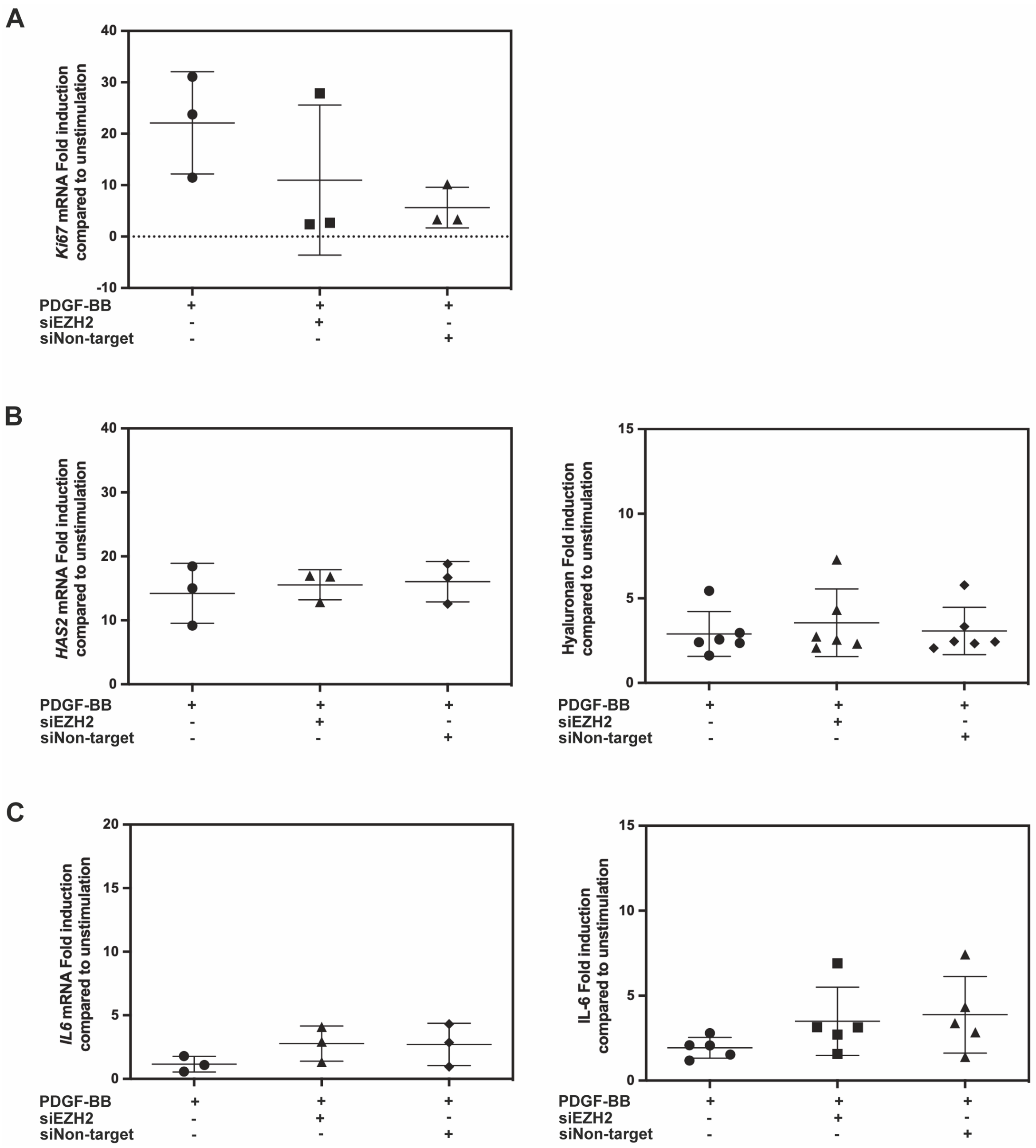


**Supplementary figure S7.**

**The effect of *EZH2* siRNA on *Ki67, HAS2* and *IL6* mRNA and hyaluronan and IL-6 expression in PDGF-BB-stimulated orbital fibroblasts.** GO orbital fibroblasts (n=3-6) were transfected with *EZH2* siRNA or non-targeting siRNA for 48 hours and then stimulated with PDGF-BB (50 ng/ml). After 24 hours of PDGF-BB stimulation, *Ki67* **(A)**, *HAS2* **(B)** and *IL6* **(C)** mRNA expression were determined by RT-PCR and normalized to the control gene *ABL*. After 48 hours of PDGF-BB stimulation, the level of hyaluronan **(B)** and IL-6 **(C)** level were determined by ELISA. Fold induction at all time points were further compared to unstimulated condition. Each dot represents the orbital fibroblast strain from one individual and horizontal bars represent the mean values ± standard deviation (SD).

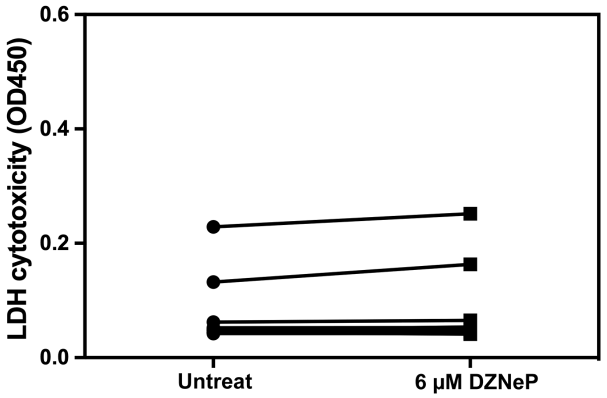


**Supplementary figure S8.**

**%Cytotoxicity of DZNeP on orbital tissues.** GO orbital tissues (n=8) were cultured with DZNeP (EZH2 inhibitor) for 24 hours, tissue supernatants were collected and measured cytotoxicity by LDH cytotoxicity assay.

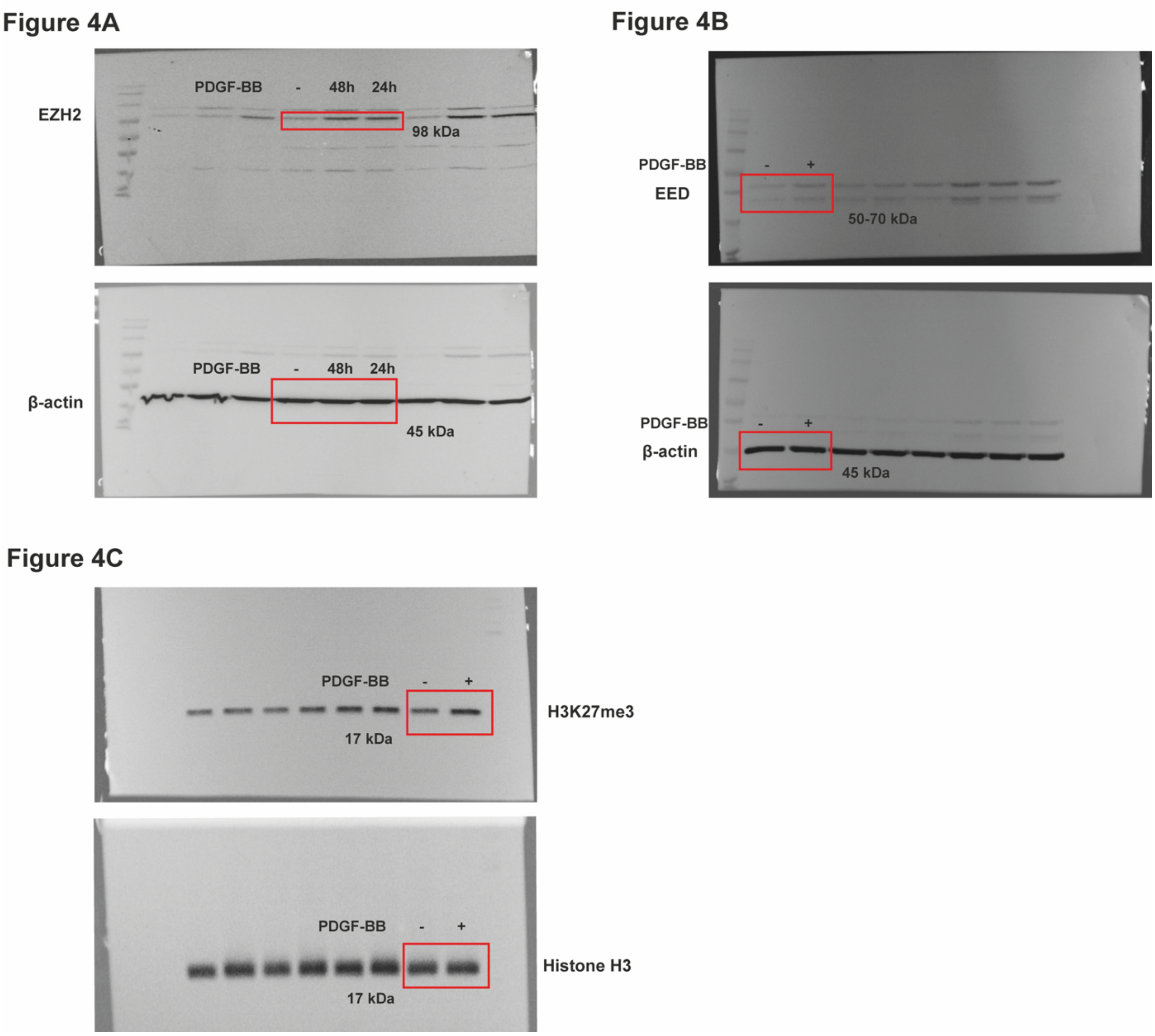


**Supplementary figure S9.**

**Uncropped blots from Figure 4A-C.** The red rectangle indicates the areas shown in Figure 4. The protein level of EZH2 **(A)**, EED **(B)** and H3K27me3 **(C)** were determined by western blot analysis in orbital fibroblasts from GO patients stimulated with PDGF-BB (50 ng/ml) for 24 and/or 48 hours.

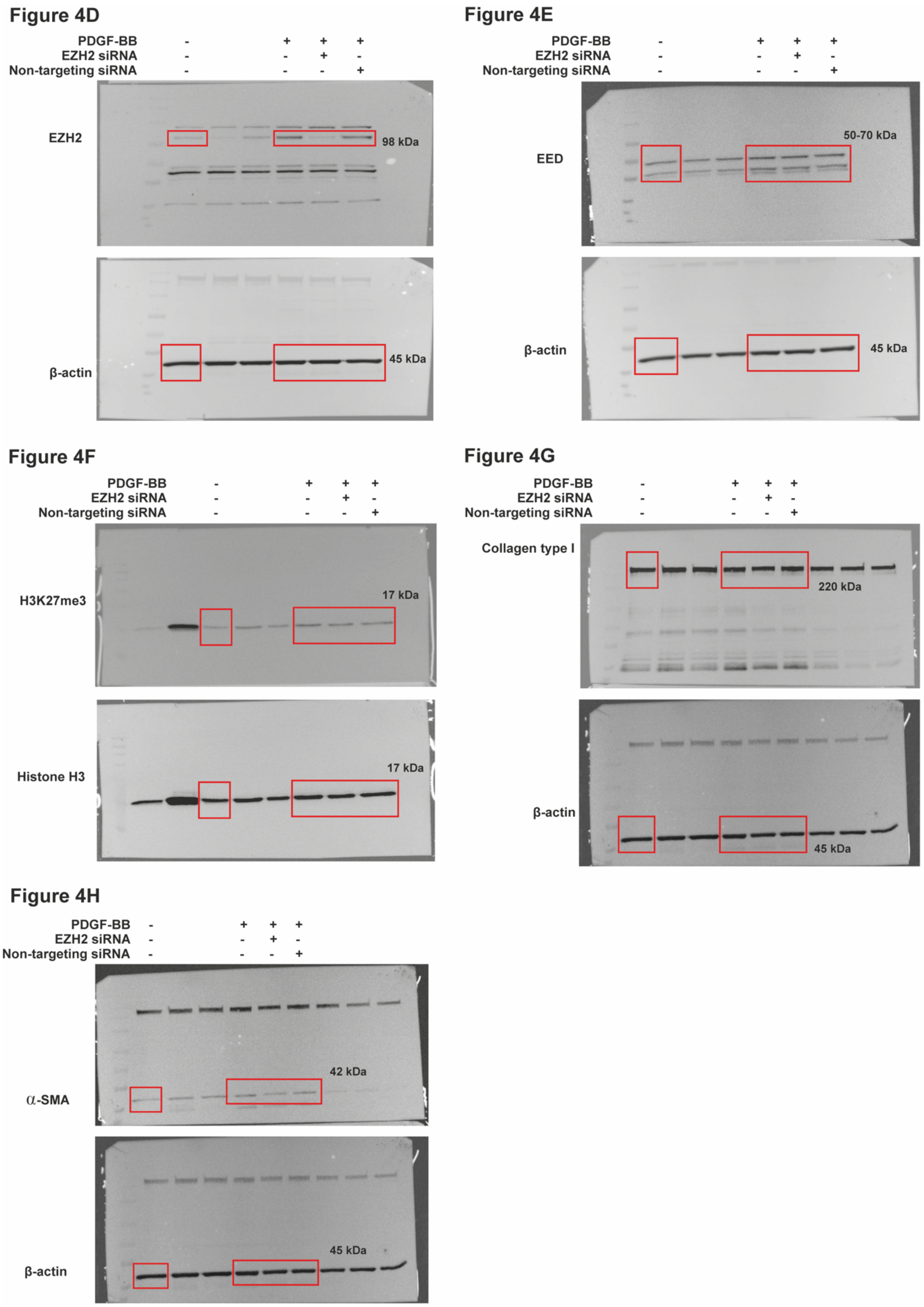

Figure 4D
PDGF-BB - + + +
EZH2 siRNA - - + -
Non-targeting siRNA - - - +
EZH2
98 kDa
β-actin
45 kDa
Figure 4E
PDGF-BB - + + +
EZH2 siRNA - - + -
Non-targeting siRNA - - - +
EED
50-70 kDa
β-actin
45 kDa
Figure 4F
PDGF-BB - + + +
EZH2 siRNA - - + -
Non-targeting siRNA - - - +
H3K27me3
17 kDa
Histone H3
17 kDa
Figure 4G
PDGF-BB - + + +
EZH2 siRNA - - + -
Non-targeting siRNA - - - +
Collagen type I
220 kDa
β-actin
45 kDa
Figure 4H
PDGF-BB - + + +
EZH2 siRNA - - + -
Non-targeting siRNA - - - +
α-SMA
42 kDa
β-actin
45 kDa

**Uncropped blots from Figure 4D-H.** The red rectangle indicates the areas shown in Figure 4. GO orbital fibroblasts were transfected with *EZH2* siRNA or non-targeting siRNA for 48 hours and then stimulated with PDGF-BB (50 ng/ml). After 48 hours of PDGF-BB stimulation, the level of EZH2 **(D)** and EED **(E)** protein expression were determined by western blot analysis and normalized to β-actin expression. The level of H3K27me3 protein expression **(F)** was determined by western blot analysis and normalized to histone H3 expression. The level of collagen type I **(G)** and $\alpha$-SMA **(H)** protein expression was determined by western blot analysis and normalized to β-actin expression.

**Supplementary fig S6C**

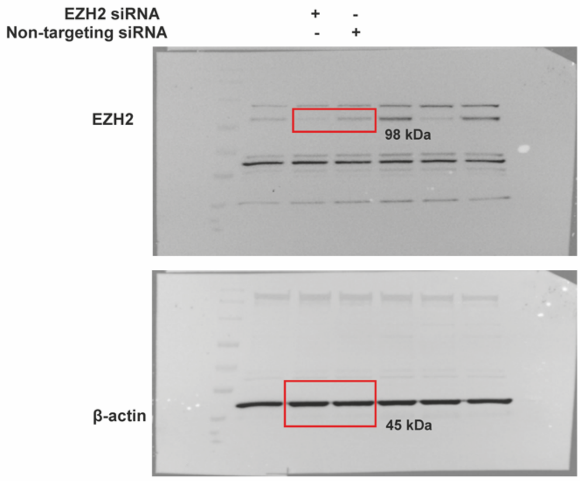


**Supplementary fig S6D**

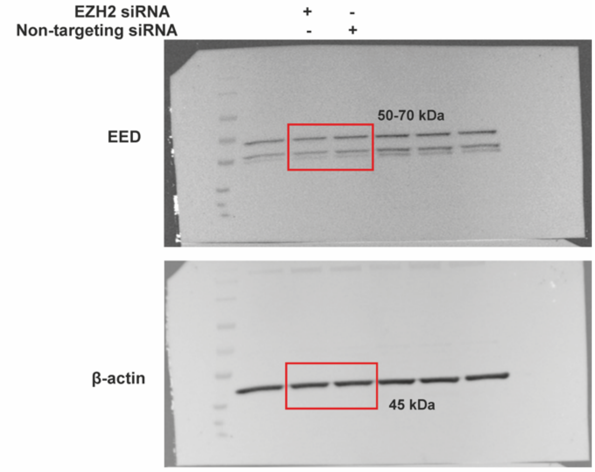


**Supplementary fig S6E**

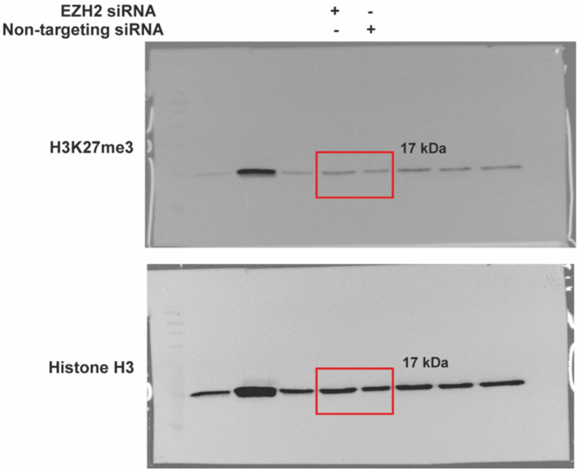


**Uncropped blots from Supplementary fig S6C-E.** The red rectangle indicates the areas shown in Supplementary fig S6C-E. GO orbital fibroblasts were transfected with *EZH2* siRNA or non-targeting siRNA for 48 hours and then the level of EZH2 **(C)** and EED **(D)** protein expression was determined by western blot analysis and normalized to β-actin expression. The level of H3K27me3 protein expression **(E)** was determined by western blot analysis and normalized to histone H3 expression.

**Supplementary Table S1: GO patients characteristics**

| GO patient | Gender | Age | CAS score | Experiment |
|---|---|---|---|---|
| I_GO_TH_4 | Male | 42 | 0 | *in vitro* |
| I_GO_TH_7 | Female | 53 | 0 | *in vitro* |
| I_GO_TH_8 | Male | 63 | 1 | *in vitro* |
| I_GO_TH_9 | Female | 59 | 0 | *in vitro* |
| I_GO_TH_11 | Male | 37 | 0 | *in vitro* |
| I_GO_TH_12 | Female | 41 | 0 | *in vitro* |
| I_GO_TH_14 | Female | 53 | 2 | *in vitro* |
| I_GO_TH_17 | Female | 58 | 0 | *in vitro* |
| I_GO_TH_25 | Male | 40 | 0 | *ex vivo* |
| I_GO_TH_26 | Male | 55 | 2 | *ex vivo* |
| I_GO_TH_27 | Male | 39 | 0 | *in vitro, ex vivo* |
| I_GO_TH_28 | Female | 60 | 2 | *ex vivo* |
| I_GO_TH_29 | Male | 40 | 0 | *ex vivo* |
| I_GO_TH_31 | Female | 30 | 0 | *ex vivo* |
| I_GO_TH_32 | Female | 30 | 1 | *ex vivo* |
| I_GO_TH_33 | Female | 48 | 1 | *ex vivo* |
| I_GO_TH_34 | Female | 27 | 0 | *ex vivo* |
| I_GO_TH_35 | Female | 30 | 0 | *ex vivo* |
| I_GO_TH_43 | Female | 63 | 0 | *in vitro* |
| I_GO_TH_51 | Male | 50 | 0 | *in vitro* |
| I_GO_TH_52 | Male | 69 | 0 | *in vitro* |
| I_GO_TH_56 | Male | 50 | 0 | *in vitro* |
| I_GO_TH_57 | Male | 47 | 0 | *in vitro* |
| I_GO_TH_58 | Female | 35 | 0 | *in vitro* |
| I_GO_TH_60 | Female | 45 | 0 | *in vitro* |

## Supplementary Table S2: Histone methyltransferase inhibitor library

| HMT enzymes | HMTs inhibitors | Highest Concentration tested (μM) |
|---|---|---|
| EZH2 | DZNeP | 6 |
| G9a | BIX 01294 | 5.4 |
| DOT1L | Pinometostat | 4.5 |
| SETD7 | PFI-2 | 1 |

**Supplementary Table S3: Primer-probe combinations**

| Gene | Forward primer (5'-3') | Reverse primer (5'-3') | Probe (5' FAM-3' TAMRA) |
|---|---|---|---|
| *ABL* | TGGAGATAACATCTAAGCATAACTAAAGGT | GATGTAGTTGCTTGGGACCCA | CCATTTTTGGTTTGGGCTTCACACCATT |
| *HAS2* | AATGGGGTGGAAAAAGAGAAGTC | CAACCATCTTCTTCTAAAAC | TCCACACTTCGTCCCAGTGCTCTGA |
| *COL1A1* | ACTGGCCCCCCTGGTCC | GGGCTCTCCAGCAGCACCTT | CCGGACCCCCAGGCCCACCT |
| *IL6* | TAGCCGCCCCACACAGA | GTGCCTCTTTGCTGCTTTCAC | AGCCACTCACCTCTTCAGAACGAATTGACA |
| *ACTA2* | CACTGCCTTGGTGTGTGACAAT | CGTAGCTGTCTTTTTGTCCCATTC | TGTTTTCCCATCCATTGTGGGACGTC |
| *Ki67* | ATTGAACCTGCGGAAGAGCTGA | GGAGCGCAGGGATATTCCCTTA | ACGACATGAAAACCAAAGA |
| *EZH2* | TaqMan® Gene Expression Assays (Hs00544830_m1) (Thermo Fisher Scientific Inc., NY, USA) | | |
| *EZH1* | TaqMan® Gene Expression Assays (Hs00157470_m1) (Thermo Fisher Scientific Inc., NY, USA) | | |
| *G9a* | TaqMan® Gene Expression Assays (Hs01053846_m1) (Thermo Fisher Scientific Inc., NY, USA) | | |
| *DOT1L* | TaqMan® Gene Expression Assays (Hs01579928_m1) (Thermo Fisher Scientific Inc., NY, USA) | | |

**Supplementary Table S4: Antibodies (Cell Signaling Technology, Inc.)**

| | |
|---|---|
| **Primary antibodies** | Ezh2 (D2C9) XP® Rabbit mAb #5246, 1:1000, RRID:AB_10694683 |
| | EED (E4L6E) XP® Rabbit mAb #85322, 1:1000, RRID:AB_2923355 |
| | COL1A1 (E8F4L) XP® Rabbit mAb #72026, 1:1000, RRID:AB_2904565 |
| | α-Smooth Muscle Actin (D4K9N) XP® Rabbit mAb #19245, 1:1000, RRID:AB_2734735 |
| | GAPDH (D16H11) XP® Rabbit mAb #5174, 1:2000, RRID:AB_10622025 |
| | Histone H3 (D1H2) XP® Rabbit mAb #4499, 1:1000, RRID:AB_10544537 |
| | Tri-Methyl-Histone H3 (Lys27) (C36B11) Rabbit mAb #9733, 1:1000, RRID:AB_2616029 |
| | β-Actin (13E5) Rabbit mAb #4970, 1:2000, RRID:AB_2223172 |
| **Secondary antibody** | mouse anti-rabbit IgG (HRP-conjugate) secondary antibody (#5127, 1:4000), RRID:AB_10892860 |
